# Editorial: Regulation of hormone and growth factor signalling by ubiquitin and ubiquitin-like protein modifications

**DOI:** 10.3389/fendo.2024.1397685

**Published:** 2024-03-22

**Authors:** Sudha K. Shenoy, Neil J. Grimsey, Robert C. Piper

**Affiliations:** ^1^ Division of Cardiology, Department of Medicine, Duke University Medical Center, Durham, NC, United States; ^2^ Department of Cell Biology, Duke University Medical Center, Durham, NC, United States; ^3^ Department of Pharmaceutical and Biomedical Sciences, College of Pharmacy, University of Georgia, Athens, GA, United States; ^4^ Department of Molecular Physiology and Biophysics, University of Iowa, Iowa City, IA, United States

**Keywords:** ubiquitination, E3 ligase, PCOS (polycystic ovarian syndrome), MAFLD (Metabolic dysfunction-associated fatty liver disease), GLUT4

Here we highlight a Research Topic of review articles that discuss recent developments in the context of “*Regulation of hormone signalling by ubiquitin and ubiquitin-like protein modifications*”. Ubiquitin (Ub) is a small protein that gets covalently attached to proteins by a highly regulated enzymatic cascade (**Figure 1**) involving three types of enzymes, Ub-activating E1, Ub-conjugating E2, and Ub-ligase E3 (1). The process of ubiquitination (or ubiquitylation) is conserved throughout eukaryotes and is used for a diverse array of cellular functions. Ub serves as a protein interaction platform that can potentially mediate a dynamic interface with a plethora of ubiquitin-interacting proteins that in turn can affect different outcomes. Ubiquitination was discovered over four decades ago as a process to mark proteins for rapid degradation (2), which is mediated by the 26S proteasome which has an expansive capacity to non-covalently bind Ub (3). It is now appreciated that ubiquitination of proteins is a multifunctional modification that can affect not only protein half-life, but also provoke dynamic changes in protein interactions, sub-cellular localization, kinase activation, and signal propagation.

**Figure 1:**
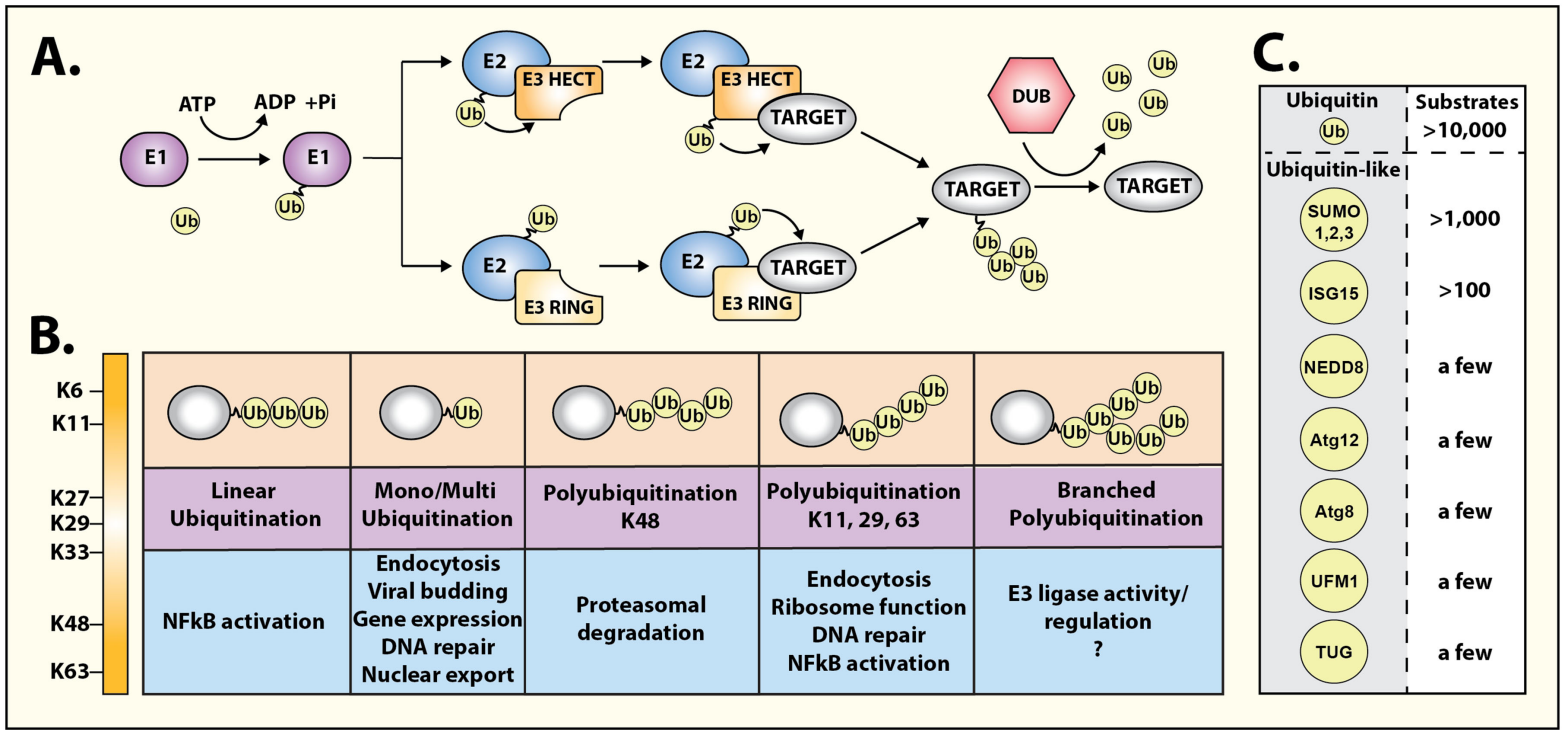
Diversity of ubiquitin and ubiquitin-like signaling. **(A)** Schematic representation of ubiquitination cascade, consisting of three types of enzymes: E1 ubiquitin-activating, E2 ubiquitin-conjugating, and E3 ubiquitin ligase. Two principal families of E3 ligases, namely, RING domain-containing E3 ligases or HECT domain-containing E3 ligases ubiquitinate target/substrate proteins through distinct mechanisms. Ubiquitin moieties can be removed and recycled using deubiquitinases (DUBs) to terminate ubiquitin-driven responses. **(B)** Ubiquitin modifications can take multiple forms, initiated on lysine residues (mono-ubiquitination), and can lead to sequential ubiquitination on one of the seven ubiquitin lysine residues leading to the formation of multi-, linear, poly-, or branched ubiquitination chains. Each conformation generates a specific topology enabling selective protein binding and initiation of precise functional responses. **(C)** Thousands of substrates are ubiquitinated in cells. However, a growing range of ubiquitin-like modifiers have been identified albeit their repertoire of substrates is markedly fewer than ubiquitin.

The functional diversity of ubiquitination is driven by the complex nature of when, where, and how target proteins are ubiquitinated (**Figure 1**). The proximity of ubiquitin-binding proteins or complexes throughout the cell can play an important role in the immediate outcome of protein ubiquitination. Furthermore, Ub itself can be polymerized into different topologies, which adds to the complexity of this covalent modification of substrate proteins. Accordingly, different types of modifications of a particular substrate can lead to distinct downstream consequences. The major types of ubiquitination include monoubiquitination (single ubiquitin at one lysine), multi-monoubiquitination (single ubiquitin at multiple lysines), lysine-48 polyubiquitination (ubiquitin chains formed by linkage at lysine 48 in ubiquitin), lysine-63 polyubiquitination (ubiquitin chains formed by linkage at lysine 63 in ubiquitin) or linear ubiquitination (ubiquitin chains formed by linkage at the amino terminus) (**Figure 1**) (4–6).

Ubiquitin is not alone in its task of orchestrating cellular pathways, and about a dozen ‘ubiquitin-like’ proteins have been discovered that are structurally related to Ub and employ similar mechanisms for covalent attachment to substrate proteins (**Figure 1**). An additional layer of regulation is bestowed by deubiquitinases or enzymes that remove Ub and terminate events initiated by ubiquitination. ~100 deubiquitinases are expressed in mammalian cells that act in a substrate-specific manner with also the capacity to distinguish different Ub topologies (7). Protein ubiquitination can be considered a complex code by which intracellular communication is granted specificity or is fine-tuned. This has a pronounced effect on many signal transduction modules as has been shown for growth factor receptors and for members of the large family of heptahelical G protein-coupled receptors (4, 8, 9). The four articles included in this Research Topic underscore the widespread impact of ubiquitination in regulating diverse biological pathways and its relevance to human disease.

The first article by Tang et al., discusses the role of Ub in regulating receptor tyrosine kinases (RTKs), which are single-pass transmembrane receptors that are activated by growth factors. RTKs including epidermal growth factor receptor, platelet-derived growth factor receptor, fibroblast growth factor receptor, and hepatocyte growth factor receptor are ubiquitinated by the Casitas B-lineage lymphoma (Cbl) family of E3 ubiquitin ligases. The tyrosine kinase binding domain in Cbl proteins (c-Cbl, Cbl-b and Cbl-3) binds to phosphorylated motifs of activated RTKs leading to ubiquitination of the RTKs. This attenuates growth factor signalling by promoting lysosomal trafficking and degradation of activated RTKs. Cbls are Ub-ligases(E3) of the RING (really interesting new gene) family and engage specific Ub-conjugating (E2) enzymes and adaptors to ubiquitinate their RTK substrates. Cbl ligases are proto-oncogenes that when mutated, enhance RTK signalling, which can also be compromised by alternate mechanisms when cells have normal Cbl activity. Boosting the activity of Cbls to suppress RTK signalling and cancer growth is a potential therapeutic approach, although this strategy faces many challenges due to off-target effects on additional substrates that are linked with Cbls. Nonetheless, an expansion of our understanding on Cbl-mediated ubiquitination and downregulation of RTKs, can lead to the development of new ideas for drug-targeting the elevated growth factor receptor activity in cancer cells.

In the second article, Bogan et al., describe in great depth how the ubiquitin-like processing of Tether Containing UBX Domain for GLUT4 (TUG) regulates glucose uptake and energy metabolism. TUG binds to the glucose transporter GLUT4-containing vesicles, and retains them intracellularly, sequestering GLUT4 from the plasma membrane. Insulin induces proteolytic processing of TUG by Usp25m protease in fat and muscle cells, which generates a ubiquitin-like protein modifier TUGUL, which is then covalently attached to the cellular motor protein called kinesin KIF5B. TUGULated KIF5B mediates the movement of GLUT4 vesicles to the cell surface, allowing the delivery of GLUT4 to the plasma membrane to promote glucose uptake. By regulating GLUT4 trafficking, TUG plays an important role in glucose homeostasis, and perhaps in regulating insulin sensitivity. Interestingly, the cleaved C terminal domain of TUG translocates to the nucleus and enables gene transcription to promote lipid oxidation and thermogenesis. Although TUG does not bear sequence homology with ubiquitin, TUG and its proteolytic processing represent a novel paradigm that mimics ubiquitination of proteins.

The article by Wei et al. compares ubiquitination along with other well-studied post-translational modifications (phosphorylation, methylation, and acetylation) for their impact on polycystic ovary syndrome (PCOS), a chronic metabolic, reproductive, and psychiatric disorder that affects females. Recent studies that evaluated the transcriptome of granulosa cells and peripheral blood mononuclear cells found genes of the ubiquitination pathway to be enriched among the differentially expressed genes correlating with PCOS. Additionally, there is an emerging theme on the relationship between expression of ovulation genes and hormone regulation as well as ubiquitination of androgen receptor. Although research linking ubiquitination and PCOS is still at infancy, the findings so far point to the possibility of leveraging ubiquitin pathway components toward therapeutic targeting for PCOS.

The article by Zhang et al. in this Research Topic summarizes the role of RING-domain Ub-ligases(E3) of the tripartite motif (TRIM) family in metabolic-associated fatty liver disease (MAFLD). Of the 80 members of the TRIM family of Ub-ligases, roughly 12 have been correlated with Ub-mediated regulation of various MAFLD-related proteins and progression of liver disease. For example, TRIM8 expression is specifically induced in the liver during obesity and correlated with inflammation and dyslipidemia, wherein TRIM8 promotes pathological signalling through the kinase TAK1. Severe MAFLD also correlates with elevated expression of TRIM6, TRIM9, TRIM22, TRIM59 and TRIM69. The authors present a comprehensive summary of TRIM-mediated regulation of MAFLD, hepatic role in insulin resistance, and liver cancer, emphasizing the need to fully understand the complex and heterogeneous roles of TRIMs in liver disease.

The presented articles shed light on the multifaceted ways in which Ub and Ub-like proteins orchestrate diverse physiological pathways. Nearly every cellular protein undergoes Ub modification during its lifespan. And most biochemical pathways are shaped by their interaction by the highly regulated, diverse, and intricate process of ubiquitination. The dysregulation of protein ubiquitination emerges as a common element connecting various disease pathologies. Understanding the complexities of ubiquitination and how Ub and Ub-like modifications alter cellular processes at the molecular level can lead to new drugs to treat diseases spanning from hypertension, heart failure, and neurodegeneration to obesity and cancer.

## Author contributions

SS: Writing – review & editing, Writing – original draft. NG: Writing – review & editing. RP: Writing – review & editing.
